# Recent changes in the mutational dynamics of the SARS-CoV-2 main protease substantiate the danger of emerging resistance to antiviral drugs

**DOI:** 10.3389/fmed.2022.1061142

**Published:** 2022-12-14

**Authors:** Lena Parigger, Andreas Krassnigg, Tobias Schopper, Amit Singh, Katharina Tappler, Katharina Köchl, Michael Hetmann, Karl Gruber, Georg Steinkellner, Christian C. Gruber

**Affiliations:** ^1^Innophore GmbH, Graz, Austria; ^2^Institute of Molecular Biosciences, University of Graz, Graz, Austria; ^3^Austrian Centre of Industrial Biotechnology, Graz, Austria; ^4^Field of Excellence BioHealth, University of Graz, Graz, Austria

**Keywords:** SARS-CoV-2, COVID-19, M^pro^, main protease, viral evolution, drug resistance, nirmatrelvir, Paxlovid

## Abstract

**Introduction:**

The current coronavirus pandemic is being combated worldwide by nontherapeutic measures and massive vaccination programs. Nevertheless, therapeutic options such as severe acute respiratory syndrome coronavirus 2 (SARS-CoV-2) main-protease (M^pro^) inhibitors are essential due to the ongoing evolution toward escape from natural or induced immunity. While antiviral strategies are vulnerable to the effects of viral mutation, the relatively conserved M^pro^ makes an attractive drug target: Nirmatrelvir, an antiviral targeting its active site, has been authorized for conditional or emergency use in several countries since December 2021, and a number of other inhibitors are under clinical evaluation. We analyzed recent SARS-CoV-2 genomic data, since early detection of potential resistances supports a timely counteraction in drug development and deployment, and discovered accelerated mutational dynamics of M^pro^ since early December 2021.

**Methods:**

We performed a comparative analysis of 10.5 million SARS-CoV-2 genome sequences available by June 2022 at GISAID to the NCBI reference genome sequence NC_045512.2. Amino-acid exchanges within high-quality regions in 69,878 unique M^pro^ sequences were identified and time- and in-depth sequence analyses including a structural representation of mutational dynamics were performed using in-house software.

**Results:**

The analysis showed a significant recent event of mutational dynamics in M^pro^. We report a remarkable increase in mutational variability in an eight-residue long consecutive region (R188-G195) near the active site since December 2021.

**Discussion:**

The increased mutational variability in close proximity to an antiviral-drug binding site as described herein may suggest the onset of the development of antiviral resistance. This emerging diversity urgently needs to be further monitored and considered in ongoing drug development and lead optimization.

## 1 Introduction

The Coronavirus Disease 2019 (COVID-19) pandemic, caused by severe acute respiratory syndrome coronavirus 2 (SARS-CoV-2), is being combated worldwide by non-therapeutic measures and massive vaccination programs. Still, therapeutic options such as the SARS-CoV-2 main-protease (M^pro^, also referenced as 3C-like protease or NSP5) inhibitor nirmatrelvir/ritonavir (sold under the brand name Paxlovid™) (1) are absolutely required due to the progressive evolution toward escape from natural or induced immunity (2–4), generally driven by mutations within the spike protein (5).

As mutation rates are estimated to be moderate to high in coronaviruses (6), treatment options readily adaptable to virus variants or unaffected by spike-protein mutations are an important piece of the puzzle to reduce the threat of the virus, not just to the unvaccinated portion of the population (7). In the future, additional SARS-CoV-2 variants will most probably emerge that evade the immune system, as it was, for instance, the case with the Beta (B.1.351), Gamma (P.1), and recently the Omicron (B.1.1.529) (8–10) variants, with the latter driving the formation of the current waves (11–13).

Because of the importance of the spike protein’s genetic drift (14), efforts are being made to closely monitor and track changes relevant to infectiousness (15, 16) and immunity (17, 18). However, since drug-discovery efforts targeting M^pro^ (19) had already begun as early as January 2020, computational models and experimentally determined protein structures needed to identify covalent (20, 21) and non-covalent inhibitors (22, 23) were and are mainly based on the “wild-type” version of M^pro^ as it was present in the first sequenced SARS-CoV-2 strain (24). This highly characterized protease plays a crucial role in the viral replication cycle as it cleaves the polyproteins pp1a and pp1ab into individual, active proteins (25). Combined with the fact that there are no known human proteases with identical cleavage-site specificity (26), which reduces unwanted side effects of drugs, this has made M^pro^ a popular target for drug development. Furthermore, Pfizer’s recent M^pro^ analyses demonstrated high sequence and structural conservation prior to the widespread use of nirmatrelvir. Still, the authors emphasized the importance of continuous genetic surveillance of M^pro^ due to the risk of drug-resistance development (27).

In order to investigate evolutionary changes in the virus genome, one can monitor the appearance of new SARS-CoV-2 isolates in relevant sequence databases such as GISAID (28). A common approach is the analysis of sequence entropy (29), which is based on Shannon’s mathematical theory of communication (30). Shannon entropy is calculated for the consensus sequence of a set of proteins. Also, it represents the frequency of mutations, allowing detection of amino-acid replacements showing a relatively high abundance. However, such an analysis cannot properly capture rare mutational events occurring in a relatively small portion within a large set of sequences (31, 32). To identify these rare mutational events, it is instrumental to track the unique amino-acid exchanges (and other changes such as insertions or deletions) and their first occurrences at certain positions in a protein. More precisely, a unique amino-acid exchange at a given position is defined as the amino acid present in the wild type being exchanged for exactly one other specific amino acid occurring at least once in the sequence data set.

Inhibition of M^pro^ by nirmatrelvir, targeting the active site of the protease, has been shown to be unaltered for the six strongly prevalent mutations G15S, T21I, L89F, K90R, P132H, and L205V (33), which came up prior to the authorization of Paxlovid™ for conditional or emergency use in December 2021. However, the ability of SARS-CoV-2 M^pro^ to develop resistances under the selective pressure of protease inhibitors has been shown *in vitro* as described in several studies available as preprints (34–36). In order to monitor mutational behavior of SARS-CoV-2, including detection of therapeutically relevant amino-acid exchanges and possible responses to environmental changes, as for instance the deployment of antivirals, we have developed a workflow that, among other effects, detects changes in mutational dynamics in critical protein regions, such as the active site of M^pro^, by a time-resolved screening of millions of publicly available genomes. Most strikingly, an in-depth analysis, considering rare mutational events, revealed accelerated mutational dynamics in close proximity to the active site of M^pro^ since the beginning of December 2021. Increased mutational variability at a common antiviral-drug binding site may suggest the onset of the development of antiviral resistance. Here we extensively discuss the effect observed in M^pro^ and briefly offer a possible interpretation for our findings.

## 2 Materials and methods

### 2.1 Processing of SARS-CoV-2 genomic data

SARS-CoV-2 genomic data was downloaded from GISAID (28) as a multiple-sequence alignment (MSA) in FASTA format (msaCodon_0630.fasta) containing 10.5 million genome sequences available by 30 June 2022. Protein sequences of the 28 SARS-CoV-2 proteins with a collection date starting from 24 December 2019 were parsed from the MSA according to their position in the reference genome sequence NC_045512.2 (Table 1) and translated employing the Biopython (37) package Bio.SeqIO. The number of protein sequences was reduced to a set of unique sequences, i.e., containing one representative for sequences with identical amino-acid compositions. Each unique sequence was aligned to the protein sequence of the reference NC_045512.2 using the Biopython (37) package Bio.pairwise2 module with a gap-opening penalty of −10 and a gap-extension penalty of −8.

**TABLE 1:** Positions of the 28 proteins within the NCBI reference genome sequence NC_045512.2.

Protein	Position (nt)	Protein	Position (nt)	Protein	Position (nt)
NSP1	266-805	NSP11	13442-13480	M	26523-27191
NSP2	806-2719	NSP12	13442-13468| 13468-16236	NS6	27202-27387
NSP3	2720-8554	NSP13	16237-18039	NS7a	27394-27759
NSP4	8555-10054	NSP14	18040-19620	NS7b	27756-27887
NSP5	10055-10972	NSP15	19621-20658	NS8	27894-28259
NSP6	10973-11842	NSP16	20659-21552	N	28274-29533
NSP7	11843-12091	Spike	21563-25384	NS9b	28284-28577
NSP8	12092-12685	NS3	25393-26220	NS9c	28734-28955
NSP9	12686-13024	E	26245-26472	NS10	29558-29674
NSP10	13025-13441				

Genome positions are given in nucleotides (nt). The pipe symbol indicates a translational frameshift.

For our analysis we only included amino-acid exchanges that were located within high-quality regions of the alignment. More precisely, exchanges adjacent to deletions, insertions, or uncertain residues (indicated with “X” in the sequence) were neglected in order to ensure the correct mapping of an exchange to its position within the sequence. Mutations not directly adjacent to deletions, insertions, or “X” were neglected if the surrounding region of 10 residues on each side of the potential exchange showed more than 50% mismatches to the wild-type sequence in combination with at least one deletion, insertion, or “X.”

Time-resolved analysis of mutation events was performed by assigning the collection dates of genome sequences retrieved from GISAID (28) to the respective protein sequences. The data set contained 253,264 genome sequences with incomplete collection dates, e.g., 2021-00-00 or 2021-03-00, which were consequently excluded from our analysis. The Supplementary material contains a flowchart that illustrates the methodology used in this work (Supplementary Figure 1).

### 2.2 Time- and in-depth sequence analysis

A python workflow was used to combine and compile sequence-collection information with mutation lists as we extracted them from the MSA file. Dates of first occurrences of unique amino-acid exchanges were identified by comparing all dates for any particular unique amino-acid exchange at a given position in a given protein. The results were saved to disk for further analysis and as input for visualization.

**FIGURE 1:**
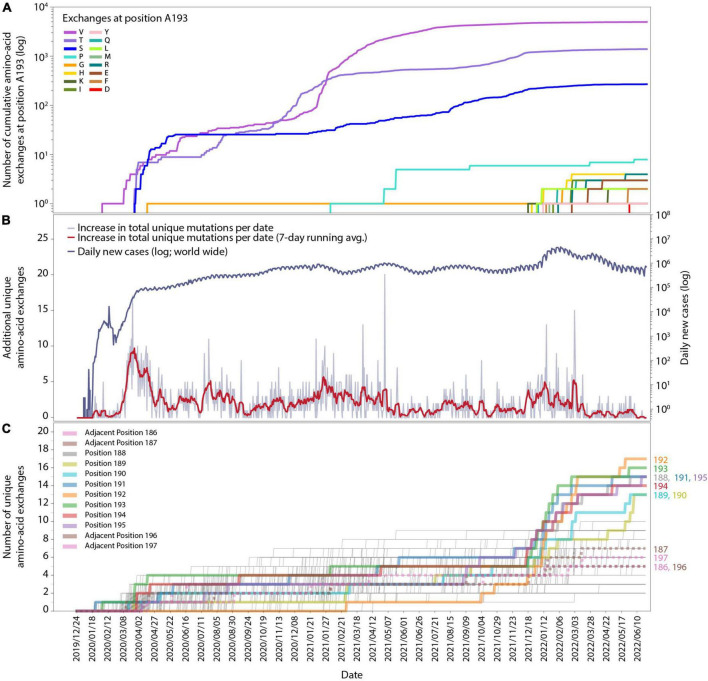
Time-resolved mutation dynamics of M^pro^. **(A)** Logarithmic abundance of single-point amino-acid exchanges at position A193, as a representative for the residues in the region of interest, within the total set of 10.5 million M^pro^ sequences. **(B)** Logarithmic daily infection numbers (dark blue) reported by WHO (https://covid19.who.int/data, accessed on 30 June 2022) in connection with the increase in total (summed over all positions and possible exchanges) unique amino-acid exchanges (gray), with a 7-day running average in red. **(C)** Number of unique amino-acid exchanges for all 306 positions in the M^pro^ sequence. Positions R188-G195 (solid) and adjacent residues V186, D187, T196, and D197 (dotted) are highlighted with bold lines.

**FIGURE 2:**
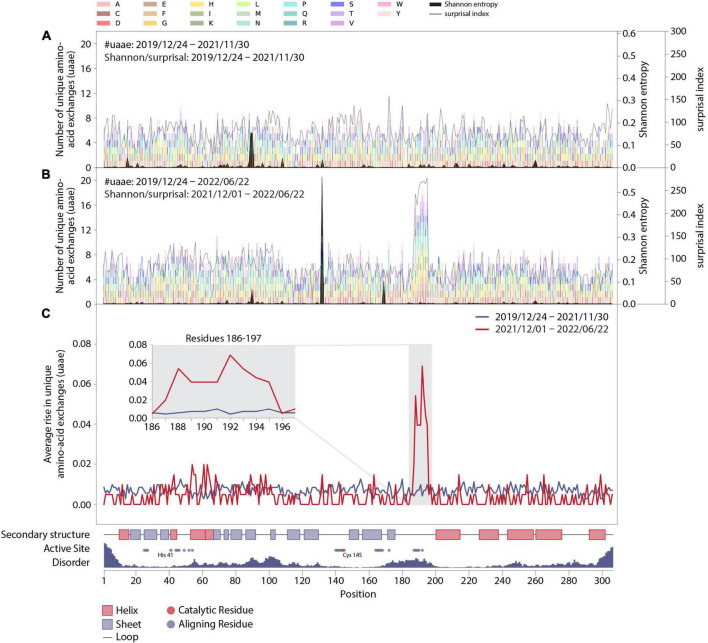
Mutation dynamics in the M^pro^ amino-acid sequence. **(A)** Final distribution of unique amino-acid exchanges for every position within the wild-type M^pro^ sequence including all sequences with a corresponding collection date before December 2021 (transparent multi-colored bars). Associated surprisal indices and Shannon entropies are superimposed in light and dark gray, respectively. **(B)** As in panel **(A)**, including all sequences with corresponding collection dates up until 22 June 2022, with associated surprisal indices and Shannon entropies, which were calculated from sequences collected from 1 December 2021 to 22 June 2022. **(C)** Average rise in unique amino-acid exchanges along the protein sequence including sequences collected before (blue) and starting from (red) 1 December 2021. Insert: Zoom in on positions R188-G195 and adjacent residues V186, D187, T196, and D197. The sequence position, complemented by structural properties partly obtained from PDB entry 7SI9 (43), is displayed on the *x*-axis.

Visualizations in Figures 1, 2 were created using the Matplotlib (38) package in Python employing standard plotting tools. Composite figures were combined from individual visualizations in post-processing.

### 2.3 Shannon entropy (H) and surprisal analysis (S)

The conservation and variability across aligned protein-sequence sites are computed by two approaches, (1) frequency-based and (2) amino-acid’s physicochemical-property-based scoring methods (39, 40). For assessing the amino-acid variability across important biochemical motifs like catalytic sites, the frequency-based scoring functions perform better than methods based on physicochemical properties (39). Therefore, we opted for Shannon-entropy, unique amino-acid-exchange, and surprisal analysis which belong to frequency-based methods. In particular, Shannon entropy is a standard tool to measure the uncertainty in a probability distribution (29, 30, 41). For quantifying the fewer populated events in a frequency distribution, surprisal analysis has the corresponding meaning, namely, the lower the probability of an event, the higher the surprisal index (30, 42). H is the Shannon entropy and S is the surprisal index in Equation 1, both calculated for each position in the M^pro^ sequence.


(1) $$\begin{array}{c} H_{i}=-\sum_{j=1}^{N}P_{i,j}\log_{2}\left(P_{i,j}\right)\\ S_{i}=-\sum_{j=1}^{N}\log_{2}\left(P_{i,j}/P_{\left(exp\right)}\right) \end{array}$$

Pairwise alignment has been used as an input for calculating the probability P_i,j_ of each amino acid j at a position i in the sequence, while P_(exp)_ is the expected probability of an amino acid j in a protein sequence. N denotes the number of unique amino acids present at a position.

### 2.4 Structural representation of mutational dynamics in M^pro^

**FIGURE 3:**
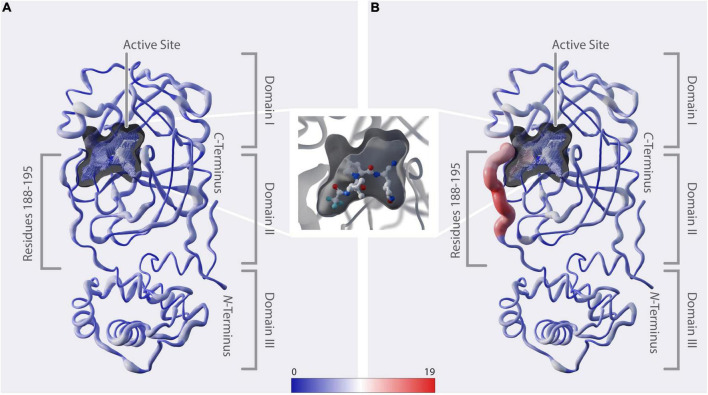
Structural representation of mutation dynamics. **(A)** Structure of M^pro^ [PDB entry 7SI9 (43)] represented as a putty figure showing the cumulative number of unique amino-acid exchanges along the protein sequence including sequences collected before 1 December 2021, referring to Figure 2A (#uaae). The active-site cavity is colored by the number of unique amino-acid exchanges of its surrounding residues (color bar). **(B)** As in panel **(A)** but including only sequences collected starting from 1 December 2021, referring to Figure 2B (#uaae). (In between panels **A, B**) Zoom in on the active-site cavity, including the inhibitor nirmatrelvir [PDB 7SI9 (43)]. Additionally, we provide a movie in the Supplementary Movie 1, showing the cumulative unique amino-acid exchanges per day from 24 December 2019 to 22 June 2022.

In order to visualize the rise in mutational variability on a 3D structural representation of SARS-CoV-2 M^pro^, the B factors within PDB entry 7SI9 (43) were exchanged with values referring to the cumulative number of unique amino-acid exchanges, as depicted in Figures 2A,B (colored bars). Structures were colored in a spectrum from blue over white to red, with a minimum and maximum value of 0 and 19, respectively. The active site cavity point cloud presented in Figure 3 was calculated using the Catalophore™ platform (44) cavity analysis and comparison program CavMan (available from Innophore GmbH)^1^ employing the LIGSITE algorithm (45) with a cutoff-value of 5 and colored by B factors. For visual representation Pymol 2.5.2 (Open Source)^2^ and Blender 3.1.2^3^ were used.

## 3 Results

In this section, we visualize the relevant data for the changes in the M^pro^ amino-acid sequence from two points of view: first, we show graphs in timeline format (Figure 1), then data as functions of the position in the M^pro^ sequence (Figure 2). Both provide important insight and are needed to understand the scope of the effect uncovered in our workflow.

We investigated the mutational dynamics of the SARS-CoV-2 M^pro^ with comparative analysis of 10.5 million SARS-CoV-2 genome sequences available at GISAID (28) by 30 June to the NCBI reference genome sequence NC_045512.2. Amino-acid exchanges within high-quality regions of these sequences were identified by pairwise alignment of 69,878 unique M^pro^ protein sequences to the wild-type protein sequence. We identified a recent and significant increase (compared to the average occurrence rate of such changes observed so far during the pandemic) in new unique mutations within a region of the M^pro^ sequence corresponding to the target site of current antiviral agents such as the protease inhibiting component of Paxlovid™, nirmatrelvir.

In total, 1,820 distinctive amino-acid exchanges were identified within the 306 residues of M^pro^, with an average number of 5.9 ± 2.1 exchanges per residue (out of 19 possible exchanges when considering the 20 canonical amino acids) until June 2022. However, a specific region of eight amino acids (residues R188-G195) revealed an unprecedented average number of 14.8 ± 1.3 exchanges per residue. A time-resolved analysis (Figure 1), based on sequence-collection dates, revealed this specific region showing a strong rise in the number of unique exchanges from the end of November 2021 (average: 5.1 ± 1.3) to end of January 2022 (average: 10.6 ± 2.0) and June 2022 (average: 14.8 ± 1.3) compared to the rest of the protein sequence (end of November 2021: 4.9 ± 1.5; end of January 2022: 5.3 ± 1.5; June 2022: 5.7 ± 1.6).

The cumulative appearance of unique amino-acid exchanges in residue A193 as a representative for the eight residues of interest (R188-G195) over time is illustrated in Figure 1A. At this particular position, exchanges from alanine to valine, threonine, serine and glycine already occurred by April 2020, followed by proline in February 2021, while exchanges to 11 other amino acids started to appear within a period of 5 months, namely from 14 December 2021 to 25 May. Such a strong quantitative increase in newly occurring amino-acid exchanges within the respective time period is observable for all eight residues of interest and absent for the adjacent residues, namely two positions down- and two positions upstream of this specific region, as can be seen in Supplementary Figures 2, 3. While the four adjacent positions V186, D187, T196, and D197 showed 3–4 unique amino-acid exchanges up to the beginning of December and gained 1–4 more unique exchanges in the time period of December 2021 until 22 June 2022, the 8 residues of interest R188-G195 showed 3–7 exchanges before and gained 8–14 exchanges after December 2021.

In order to facilitate the temporal alignment of this effect with the general development of the pandemic, we have included a timeline of the daily new cases worldwide (upper curve in Figure 1B), together with the total number of new unique amino-acid exchanges observed over time in the entire M^pro^ sequence (lower curve in Figure 1B, also see the figure caption). An analysis of new unique amino-acid exchanges per residue in the M^pro^ protein sequence over time is displayed in Figure 1C. For each position in the sequence, there is a thin line increasing by steps of one, whenever a new unique exchange appears at that position for the first time. A sudden rise at some positions can be seen starting from the beginning of December 2021, these are highlighted throughout Figure 1C as thick lines. As mentioned above, these residues are consecutive, at positions 188-195; the respective residue position is noted to the right of the panel, where each line ends. To delineate the effect better in this timeline view, we have included four more thick (but dotted) lines for two positions on each side of the eight residues of interest. Their position numbers are denoted at their lines’ ends as well.

The alignment of all three subfigures, Figures 1A–C, allows for close inspection and comparison of the temporal appearance and possible correlation of the data. While the effect visible in Figures 1A,C is clear to coincide with a global increase of infections, it is necessary to inspect positional dependence in order to better identify the effect, and to allow for possible interpretations.

In particular, to elucidate the significance of this effect, we prepared graphs allowing for observation of mutational dynamics along the sequence position of M^pro^ (Figure 2). First of all, the data shown in Figure 2 is generally divided into two parts, with the division being motivated by the effect as seen in Figure 1C and its timing, namely the changes happening in December 2021. As a result, we show data as it was collected before December 2021 and contrast it to data collected after this date. The details are the following:

In Figures 2A,B, we show an account of those unique amino-acid exchanges at each position as colored bars that we found in SARS-CoV-2 genome sequences in a specific time period. In particular, Figures 2A,B show all exchanges observed by 30 November 2021 and by 22 June 2022, respectively. The colors correspond to the different possible results of the exchange, as indicated in the legend at the top of the figure. After December 2021, the residues R188-G195 clearly stand out from the rest of the sequence, with the average number of unique amino-acid exchanges being more than doubled compared to the other residues within M^pro^.

Figures 2A,B also contain overlaid curves of results from Shannon entropy and surprisal analysis: Except for the rise of mutation P132H resulting from the emergence of variant of concern (VOC) Omicron since December 2021, no other recent and drastic cumulative evolutionary trends were unveiled in Shannon-entropy analysis, which represents the frequency of occurrence of mutations within the sequence data set. In addition to a standard sequence-entropy analysis, which involves a weight factor to scale down the randomness for each position (32), we have employed a surprisal analysis, which is more sensitive to the rare amino-acid exchanges (42) and has been used in the past to assess the complexity of viral genomes (46) and protein sequences (47, 48). Indeed, the surprisal analysis predominately highlights rare amino-acid exchanges and is more in line with the number of unique amino-acid exchanges observed at each position of the enzyme.

In order to further quantify the observed effect, we calculated the average slopes of the lines shown in Figure 1C for the two main data parts: Figure 2C shows the average slopes of the increase visible in 1C split into the date ranges before and up to 30 November 2021 (blue line), and from 1 December 2021 up to 22 June 2022 (red line). A clear distinction is visible between residues R188-G195 and the rest of the sequence, including the adjacent positions V186, D187, T196, and D197. A close-up look at these particular residues is given in the insert in Figure 2C.

Additionally, we have added structural information of the wild type M^pro^ in Figure 2 and aligned it with the residue positions: an account of the secondary structure in the protein, the position of residues aligning with the active site of M^pro^ (with catalytically active residues highlighted in red) as well as a measure of disorder along the protein sequence derived from PDB entry 7SI9 (43). Accordingly, the residues identified to be highly variable since December 2021 are within a disordered unstructured region (conceivably allowing for a number of amino-acid exchanges without deteriorating structural integrity) and comprise mainly of positions aligning to the active site of M^pro^, thus potentially influencing the binding mechanisms of certain substrates or ligands to the active site.

Considering the strong rise in new amino-acid exchanges within positions 188-195 since December 2021, which are in close proximity to the active site of M^pro^, we examined possible correlations of increasing variability in the recognition sites (the substrates) of M^pro^, namely six residues each at the *C*-terminus of NSP4, the C- and *N*-termini of NSP5-NSP15 as well as the *N*-terminus of NSP16 (49) (Supplementary Figures 6–8). Our analysis does not report a mentionable increase in the average rise of new amino-acid exchanges since December 2021 within the M^pro^ recognition sites in polyproteins pp1a and pp1ab. Additionally, we investigated the average rise of amino-acid exchanges along the protein sequences of the remaining 27 proteins within SARS-CoV-2 reference NC_045512.2 (Supplementary Figures 4–8) and found that the observed effect is in general unique for M^pro^. Nevertheless we could observe a change in mutational dynamics, although less drastically, for NS9b, nucleocapsid phosphoprotein N and membrane glycoprotein M starting from December 2021. In summary, we have found a remarkable effect in the mutational dynamics of the SARS-CoV-2 M^pro^ and described it in detail, both with regard to time-dependence as well as with regard to affected positions in its amino-acid sequence.

## 4 Discussion

The particular reasons for the sudden increase in new mutations in a specific region in the SARS-CoV-2 genome, like the rising variability at positions R188-G195 near the active site of M^pro^, which we described in detail in the previous section, are *a priori* unclear and need further investigation. The change in unique-mutation dynamics could be the result of an increased number of mutations due to an increased number of global infections, commonly referred to as a “wave” (Figure 1). Since there is no apparent reason as to why an increase in mutation frequency overall, like during a wave, would not lead to a broader increase in new unique amino-acid exchanges, the remarkable concentration of them within this particular region of eight amino acids suggests a certain recently presented selection pressure acting on this site. The few similar effects of this kind, as observed in proteins NS9b, N, and M, are collected in the Supplementary Figures 4–8 for completeness and as a starting point for more detailed investigations. Here, we will briefly offer a possible interpretation for the effect observed in M^pro^.

### 4.1 Structural implications of emerging M^pro^ variants

M^pro^ is a cysteine protease that employs a catalytic dyad consisting of C145 and H41 and comprises an attractive drug target due to its high specificity and the low toxicity of its inhibitors (26, 50, 51). The protease consists of three domains: Domain I and II show an antiparallel beta-barrel structure, while domain III, connected to domain II via a linker loop (residues 185-201), consists of a cluster of five alpha-helices. The catalytic machinery and the substrate-binding site are located in a cleft between domains I and II (52).

The 3D positional distribution of the number of unique amino-acid exchanges within genome sequences collected before December 2021 and from December 2021 up until June 2022, respectively, is depicted in Figure 3. Additionally, we provide a movie in the Supplementary Movie 1, showing the cumulative unique amino-acid exchanges per day from 24 December 2019 to 22 June 2022. The consecutive amino acids R188-G195, which we identified to comprise the recent rise in unique amino-acid exchanges, are located in the middle of the linker loop F185-T201. While unstructured regions generally allow some variability without deteriorating structural integrity, the close proximity of this specific region to the active site and its significant contribution to the dimerization, hence activity, of the enzyme (53) indicate natural restrictions on its mutability.

A vast number of M^pro^ inhibitors have been identified and evaluated preclinically as well as in clinical trials, with most of them binding to the active site (52, 54–57). Paxlovid™, with its component nirmatrelvir binding covalently to C145 (43) (Figure 3), was first approved in December 2021 and is the only protease-inhibitor authorized for conditional or emergency use against COVID-19 (58). When analyzing the active-site cavity of the enzyme, it can be observed that the residues Q189-Q192 show side-chain alignment with the cavity. In particular, Yang et al. (59) found by structural analysis of M^pro^ in complex with nirmatrelvir, that, amongst others, residues at positions 188-192 interact directly with the inhibitor and they further propose that mutations at these positions are likely to contribute to drug-resistance development. A193-G195 do not directly align to the cavity. However, considering the proximity of these residues to the binding site we assume they might play a role in substrate and inhibitor binding likewise explained by induced fit mechanism (60, 61) or when in combination with other mutations.

### 4.2 SARS-CoV-2 M^pro^ variants in the context of antiviral evasion

For decades, multidrug-resistant bacteria have been posing a severe threat. Antiviral-drug resistance is equally dangerous, in particular in the examples of resistant human immunodeficiency viruses, influenza, herpes, or hepatitis strains, which pose a serious threat to public health. Consequently, the acquisition of mutations reducing the susceptibility and clinical activity of antiviral drugs has been a perpetual hurdle in developing effective antiviral therapies (62–66). While viral evolution toward drug-escape likely comes with a somewhat reduced natural function of the viral target protein (such as the SARS-CoV-2 M^pro^), the net effect for the virus might still be advantageous.

A mutational scan of M^pro^ expressed in yeast resulted in low mutational sensitivity of residues 188-191 and 193-195, meaning that the protease allowed several amino-acid exchanges at these positions without significant loss of function, and, in contrast, high sensitivity at position 192 (67), which had been described as a mutational coldspot elsewhere (68). Our analysis of the total abundances of unique amino-acid exchanges at positions 188-195 (Supplementary Figures 2, 3) correlatively results in significantly low mutational variability at position 192 before December 2021. However, the mutational variability increased from 3 to 17 distinct amino-acid exchanges (out of 19 possible exchanges) starting from December 2021 at this position, indicating the presence of a selective pressure which allows for this large range of mutations at an otherwise conserved position. Changes in M^pro^ from Q192 to T, S, and V (which, according to our analysis, firstly occurred in December 2021, March 2022 and May 2022) resulted in decreased susceptibility to nirmatrelvir when expressed in *Escherichia coli* (69), highlighting the significance of this position in binding of the inhibitor.

It is therefore of great importance to monitor a possible global increase in the occurrence of the herein-described new unique mutations near the active site of M^pro^ (and other regions with emerging mutations) in order to be aware of the potential development of antiviral-drug resistance at an early stage. For the establishment of resilient drugs with long-term efficacy, efficient binding of the inhibitor should not rely on interaction with residues which are insensitive to mutation. For instance, as the current situation indicates, essential interactions of the inhibitor to side chains of residues R188-G195 should be avoided. Clearly, it is of equal importance to gain structural insights, examine pathways of resistance, and constantly investigate new classes of drugs to target multiple key factors of the viral infection machinery.

For coronaviruses it is still uncertain whether drug-resistant variants will persist or evolve into globally prevalent dominant variants. VOCs like Alpha, Delta, Omicron, and others are mainly characterized by new spike-RBD variants. This is most likely due to evolutionary pressure from tropism and host adaptation as well as immune- and vaccine-evasion (70), which constitutes a continuous driving force in which adapted variants with improved fitness continue to be governed by evolutionary pressure even after reproduction and jumping to the next host. Direct-acting-antiviral (DAA) evasion variants, however, will most probably behave differently: after jumping to a new host, the mutation might not always offer a fitness boost—it might even be an evolutionary disadvantage in the absence of evolutionary pressure from a DAA. An indication for such a possible disadvantage is the fact that up to the time where we saw the effect in M^pro^’s mutational dynamics, it had not occurred—not even partially. On the contrary: diversity at the positions of interest had already been stagnant over a long period of time, cf. the date range from April 2020 to November 2021 in Figure 1C.

While analyzing this data set, we have come across interesting starting points for future research, which we plan to pursue and report elsewhere. These include a quantitative model of the in-host evolutionary dynamics of coronaviruses under the pressure of a targeted antiviral drug, accompanied by structural-bioinformatic studies of M^pro^ variants in complex with its natural substrate and inhibitors. Evolutionary studies with remdesivir-treated (71) and untreated (72, 73) patients have recently been reported. For deepening the knowledge of the evolutionary process of in-host DAA evasion and the resulting impact on druggability, we further propose continuous time-resolved sequencing of SARS-CoV-2 samples collected from COVID-19 patients, who are treated with protease inhibitors, during their treatment phase. We want to clarify that there is still a possibility that the mutations reported here, although cured for high-value regions, were caused by sequencing errors. Furthermore, this article is limited to the analysis of M^pro^; a more thorough investigation of the other SARS-CoV-2 proteins belongs to the realm of future research.

## Data availability statement

Publicly available genome sequences were downloaded from https://www.gisaid.org/. An acknowledgment table including accession numbers and the origin of the processed sequences is accessible at https://doi.org/10.6084/m9.figshare.20303979. The genome sequence herein used as a wild-type reference is available at https://www.ncbi.nlm.nih.gov/nuccore/NC_045512.2. Additional supporting data is provided in the Supplementary material. The code used for the data preparation and analysis described herein is available on GitHub at https://github.com/innophore/virus.watch-mpro.

## Author contributions

LP prepared and evaluated data, performed sequence analysis, contributed to protein structure modeling and structural analysis, and drafted the manuscript with input from all authors. AK supervised data analysis, created various visualization graphs, and drafted the manuscript with input from all authors. TS assisted in data analysis. AS performed sequence analysis regarding Shannon entropy and surprisal indices. KT and KK supported the analysis of structural aspects, druggability information, and comparison to other viral models. MH and KG contributed to structural models, structural analysis, and helped interpret the structural consequences of the observed mutations. GS contributed to evaluating, preparing, and interpreting the data. CG contributed to evaluating, preparing, and interpreting data, drafting the manuscript and designed, managed, and supervised the project. All authors edited the manuscript to its final form.

## Footnotes

1
https://www.innophore.com

2
https://pymol.org

3
https://www.blender.org
