# Supplementary material for: Recent changes in the mutational dynamics of the SARS-CoV-2 main protease substantiate the danger of emerging resistance to antiviral drugs
Source: Front Med (Lausanne). 2022 Dec 14;9:1061142. doi: 10.3389/fmed.2022.1061142 (PMC9794616; doi:10.3389/fmed.2022.1061142)
Supplement: Supplementary file 1 [file Data_Sheet_1.pdf]

Supplementary Material

Recent changes in the mutational dynamics of the SARS-CoV-2 main protease substantiate the danger of emerging resistance to antiviral drugs.

Lena Parigger[1,2], Andreas Krassnigg[1], Tobias Schopper[1], Amit Singh[1,2], Katharina Tappler[2], Katharina Köchl[1], Michael Hetmann[1,2,3], Karl Gruber[1,2,3,4], Georg Steinkellner[1,2,4] and Christian C. Gruber[1,2,3,4*]

[1]Innophore GmbH, Graz, Austria
[2]Institute of Molecular Biosciences, University of Graz, Graz, Austria
[3]Austrian Centre of Industrial Biotechnology, Graz, Austria
[4]Field of Excellence BioHealth - University of Graz, Graz, Austria

*** Correspondence:**
Christian C. Gruber
christian.gruber@innophore.com

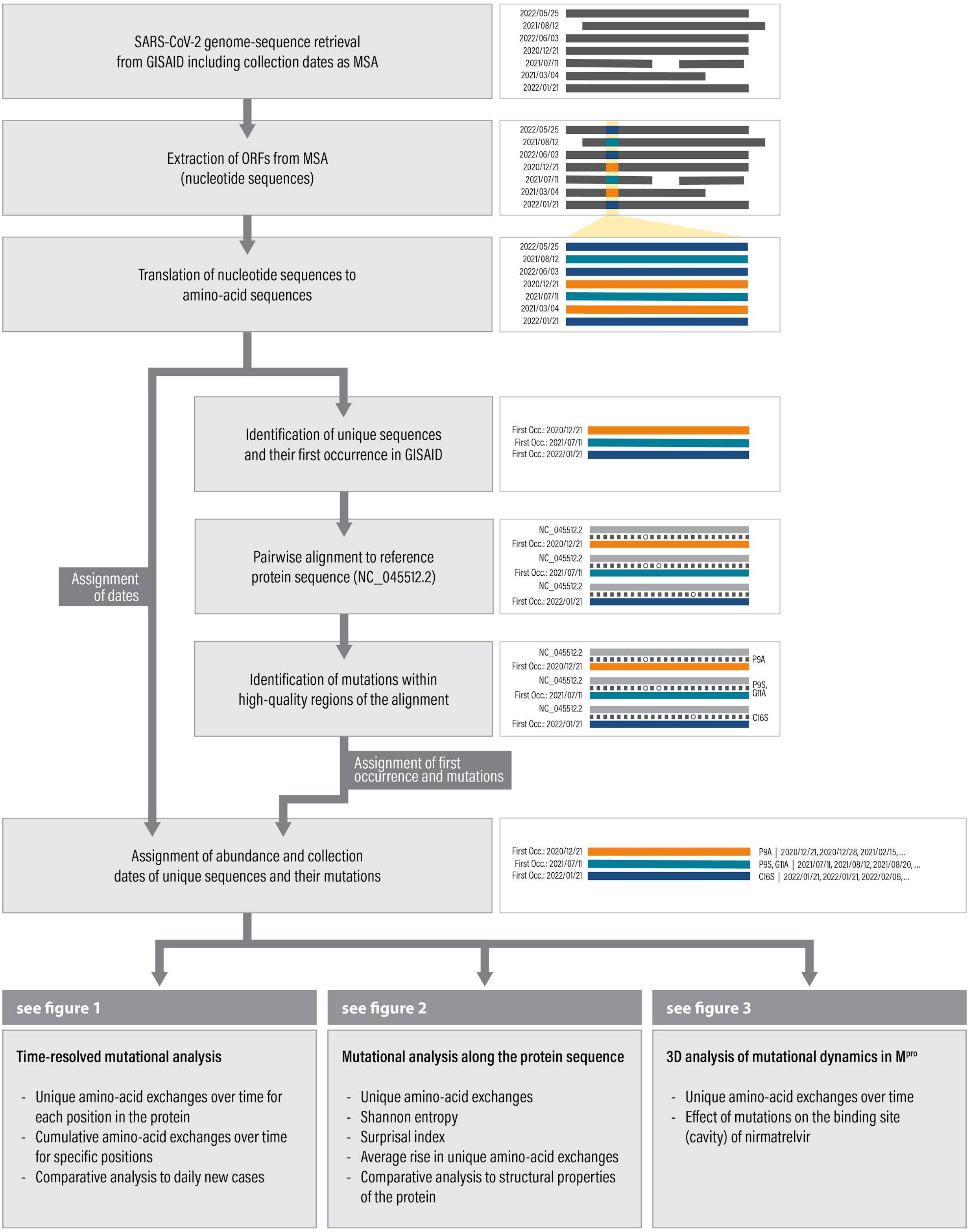

Figure S1 | A flowchart illustrating the methodology of this study. The shown dates and mutations are used for visual aspects only and do not resemble real data. MSA…multiple sequence alignment. ORFs…open reading frames.

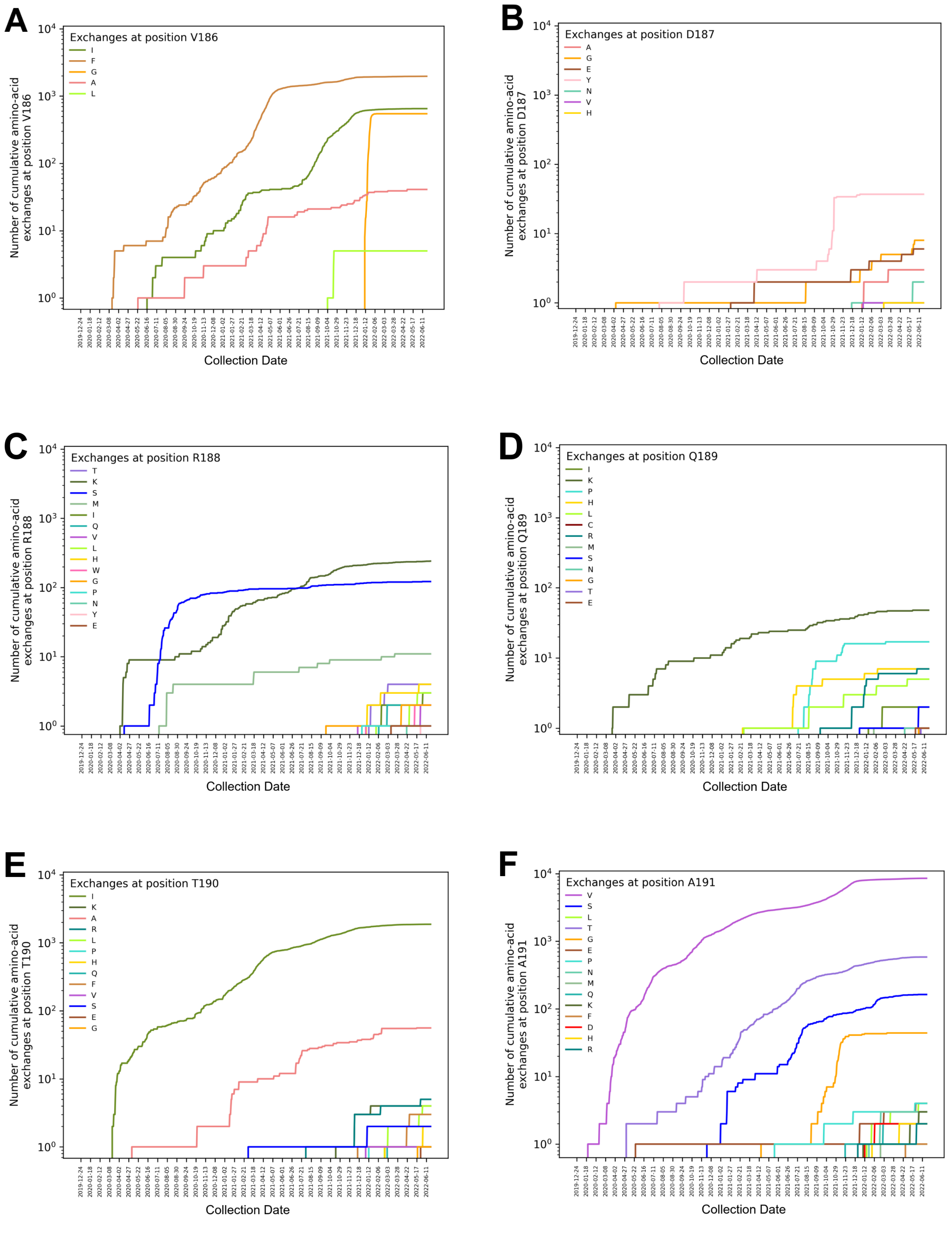

Figure S2 | Number of cumulative amino-acid exchanges at M^{pro} positions 186-191 (A-F). The logarithmic abundance of single-point amino-acid exchanges within the total set of 10.5 million M^{pro} sequences is depicted on the y-axis, with the collection date of corresponding genome sequences shown on the x-axis. The colors correspond to the different possible results of the exchange, as indicated in the legend of each figure.

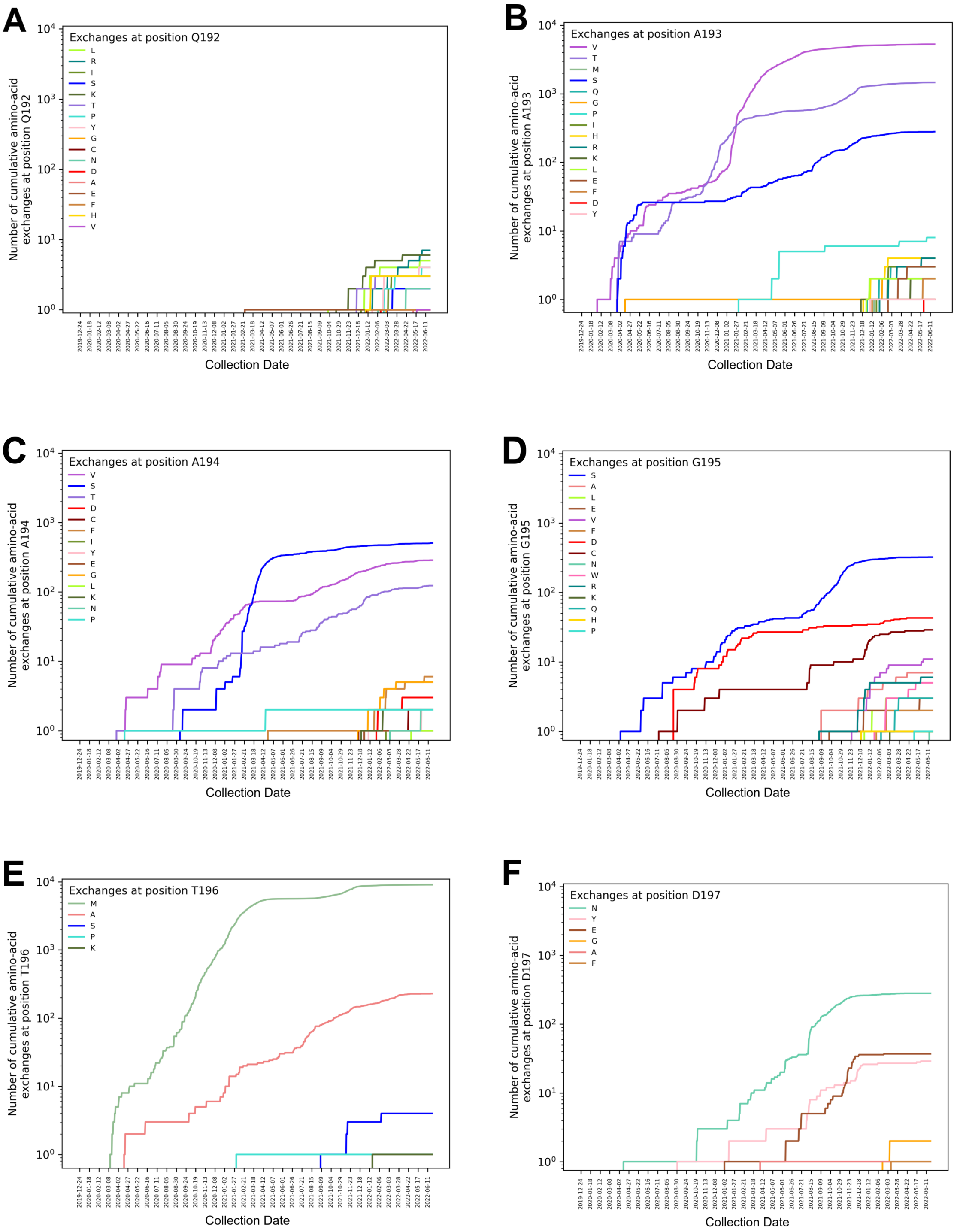

Figure S3 | Number of cumulative amino-acid exchanges at M^{pro} positions 192-197 (A-F). The logarithmic abundance of single-point amino-acid exchanges within the total set of 10.5 million M^{pro} sequences is depicted on the y-axis, with the collection date of corresponding genome sequences shown on the x-axis. The colors correspond to the different possible results of the exchange, as indicated in the legend of each figure.

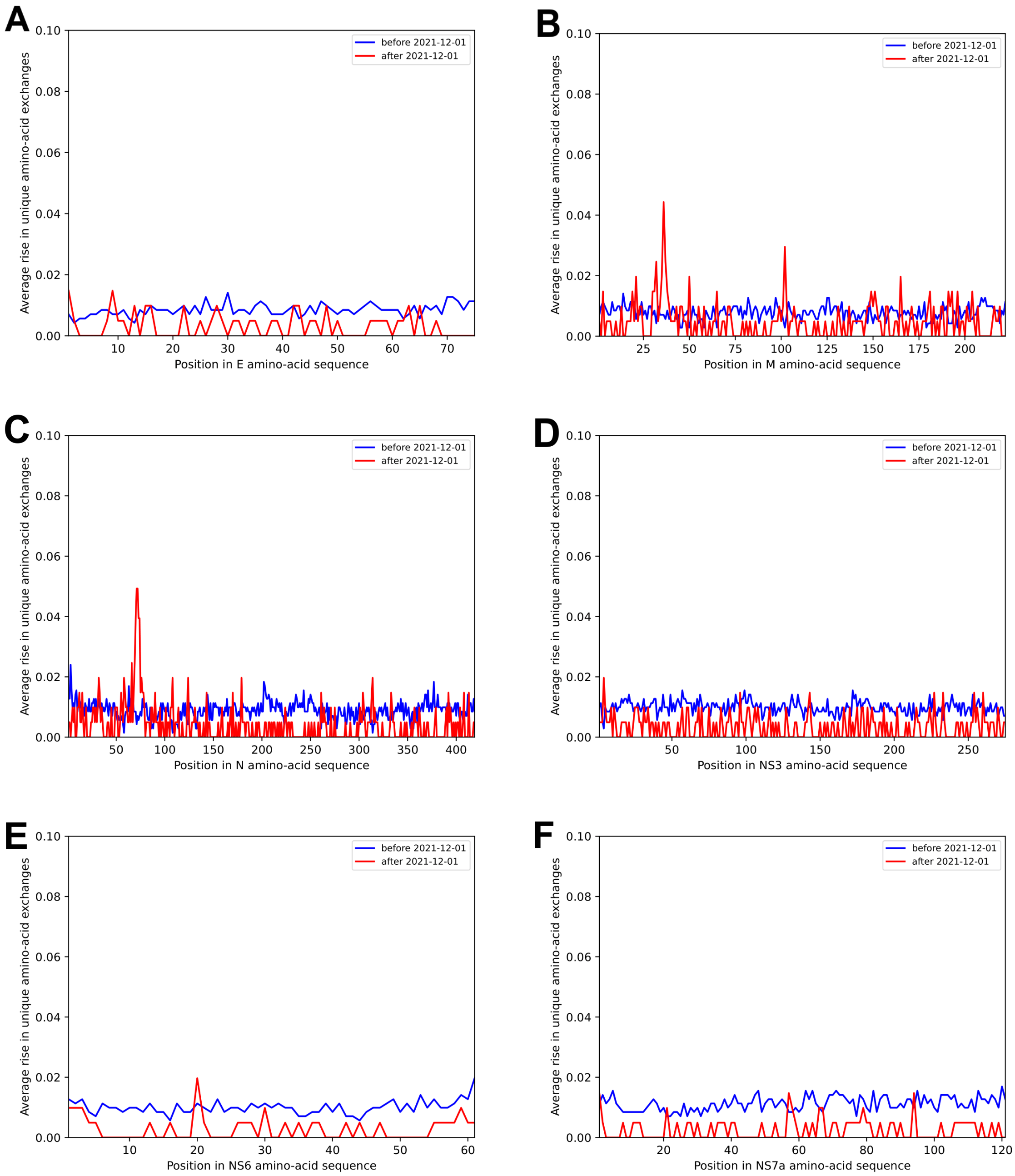

Figure S4 | Average rise in unique amino-acid exchanges in proteins E, M, N, NS3, NS6 and NS7a (A-F). The average increase in the number of unique amino-acid exchanges along the sequence of the respective proteins is split into the date ranges before and up to November 30th 2021 (blue line), and from December 1st 2021 up to June 22nd 2022 (red line).

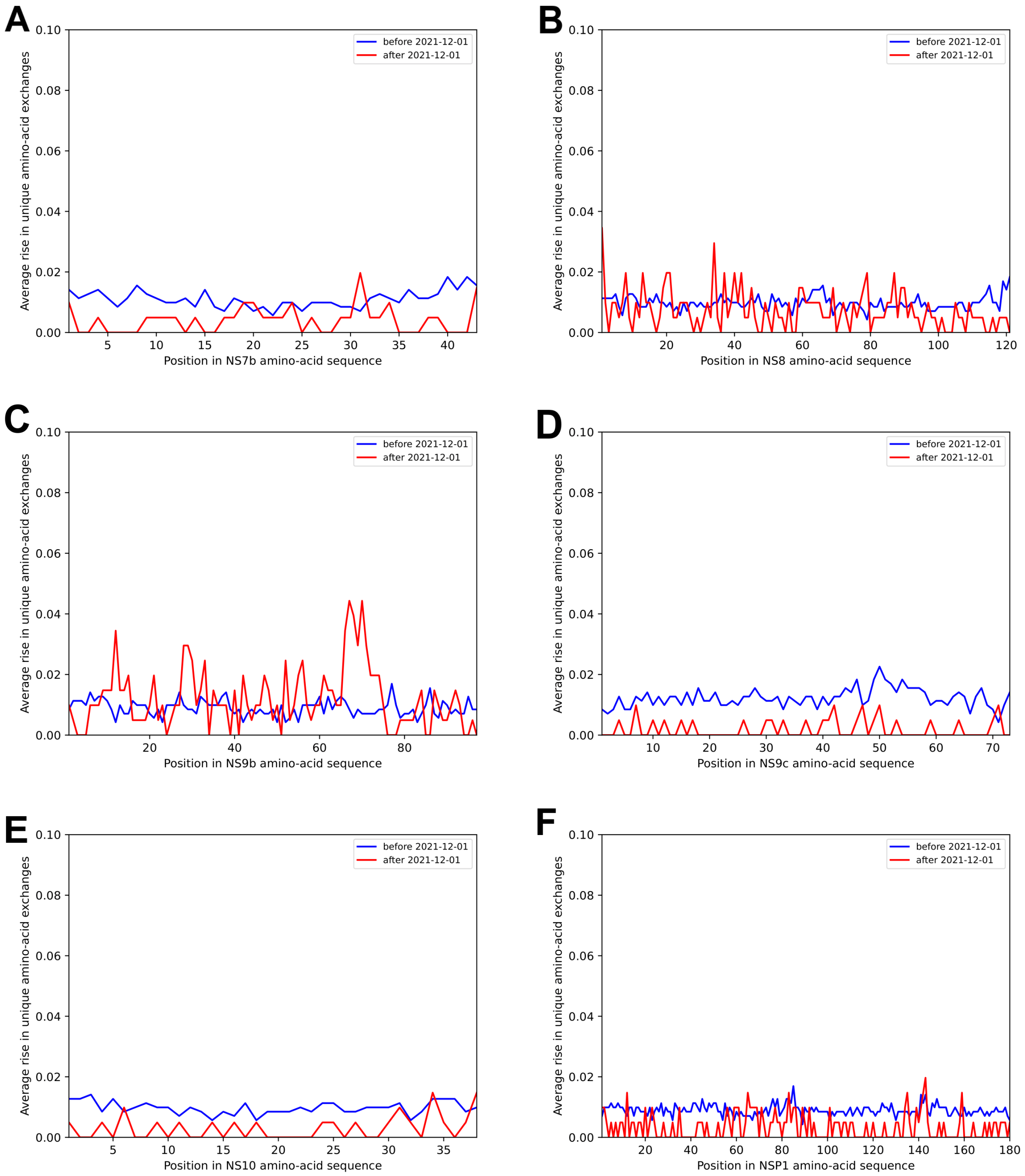

Figure S5 | Average rise in unique amino-acid exchanges in proteins NS7b, NS8, NS9b, NS9c, NS10 and NSP1 (A-F). The average increase in the number of unique amino-acid exchanges along the sequence of the respective proteins is split into the date ranges before and up to November 30th 2021 (blue line), and from December 1st 2021 up to June 22nd 2022 (red line).

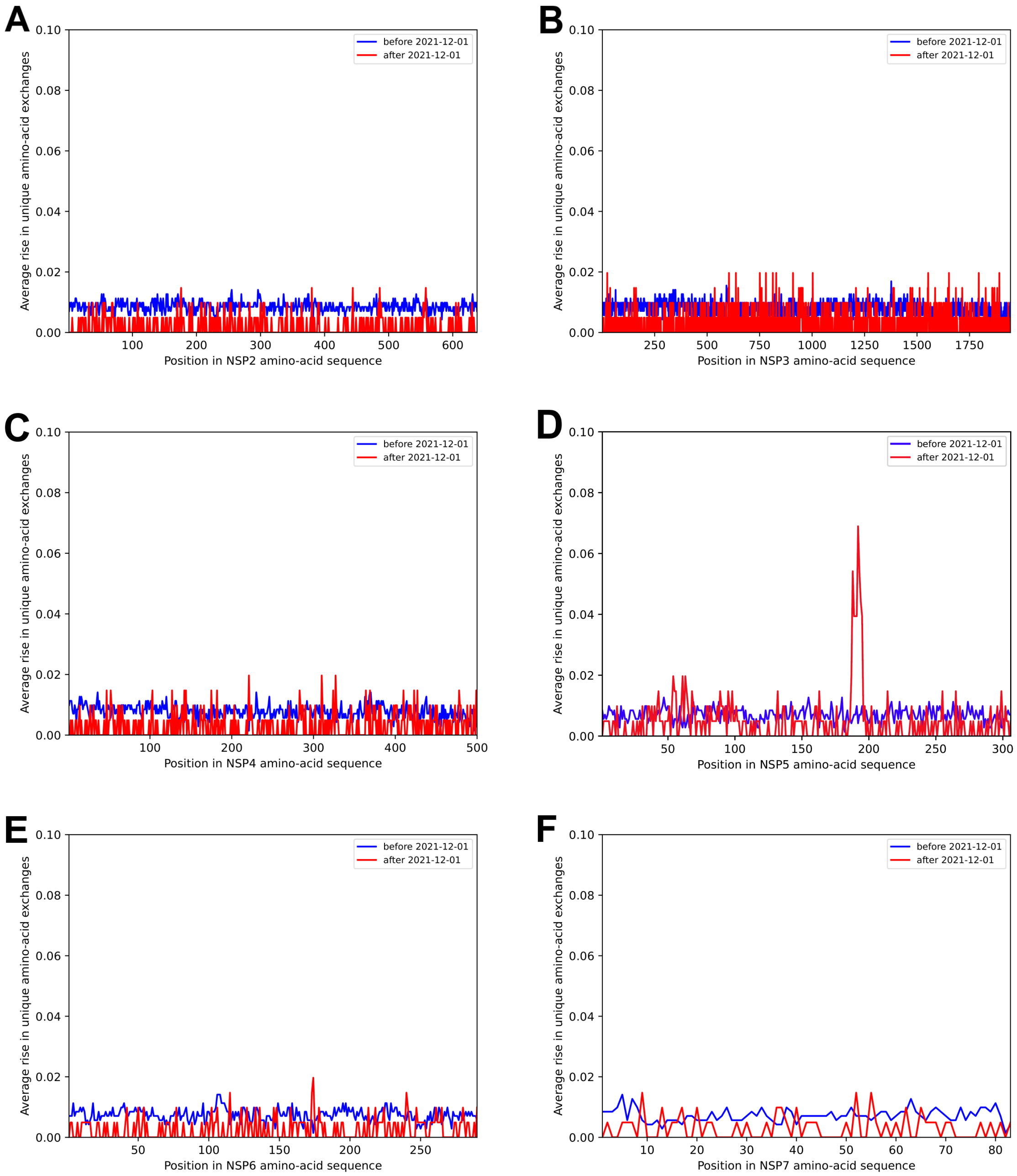

Figure S6 | Average rise in unique amino-acid exchanges in proteins NSP2, NSP3, NSP4, NSP5, NSP6 and NSP7 (A-F). The average increase in the number of unique amino-acid exchanges along the sequence of the respective proteins is split into the date ranges before and up to November 30th 2021 (blue line), and from December 1st 2021 up to June 22nd 2022 (red line).

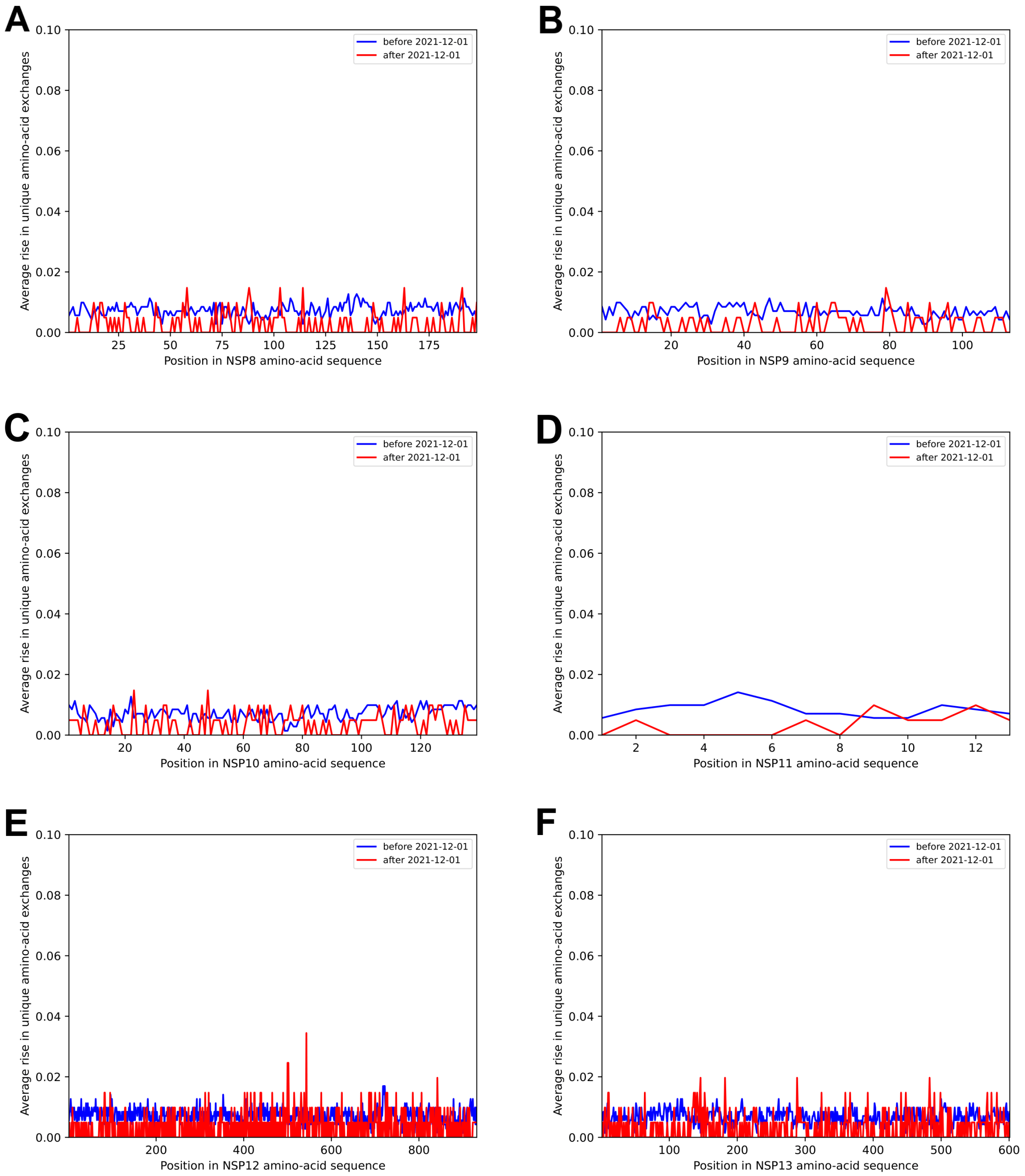

Figure S7 | Average rise in unique amino-acid exchanges in proteins NSP8, NSP9, NSP10, NSP11, NSP12 and NSP13 (A-F). The average increase in the number of unique amino-acid exchanges along the sequence of the respective proteins is split into the date ranges before and up to November 30th 2021 (blue line), and from December 1st 2021 up to June 22nd 2022 (red line).

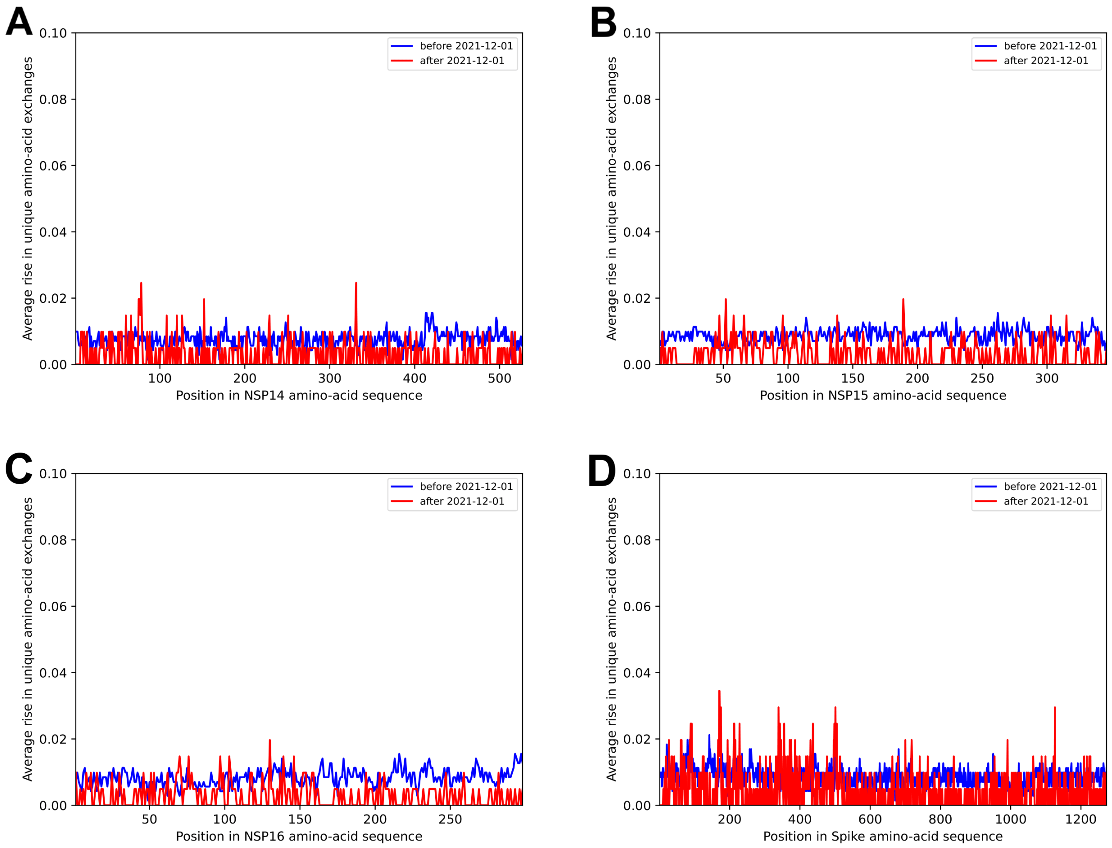

Figure S8 | Average rise in unique amino-acid exchanges in proteins NSP14, NSP15, NSP16 and Spike (A-D). The average increase in the number of unique amino-acid exchanges along the sequence of the respective proteins is split into the date ranges before and up to November 30th 2021 (blue line), and from December 1st 2021 up to June 22nd 2022 (red line).

Movie S1 (separate file) | Structural representation of mutation dynamics over time. The cumulative number of unique amino-acid exchanges per day (2019/12/24 - 2022/06/22) along the protein sequence of M^{pro} (PDB entry 7SI9(1)) is represented as a putty figure. Colors are annotated to values via the color bar in Figure 3. Nirmatrelvir, bound to C145, is shown as yellow sticks. The movie is available at https://doi.org/10.6084/m9.figshare.21118870.

SI References

1. Kneller DW, Li H, Phillips G, Weiss KL, Zhang Q, Arnould MA, Jonsson CB, Surendranathan S, Parvathareddy J, Blakeley MP, et al. Covalent narlaprevir- and boceprevir-derived hybrid inhibitors of SARS-CoV-2 main protease. *Nat Commun* (2022) 13:2268. doi: 10.1038/s41467-022-29915-z
